# A Newly Designed Curcumin Analog Y20 Mitigates Cardiac Injury via Anti-Inflammatory and Anti-Oxidant Actions in Obese Rats

**DOI:** 10.1371/journal.pone.0120215

**Published:** 2015-03-18

**Authors:** Yuanyuan Qian, Peng Zhong, Dandan Liang, Zheng Xu, Melissa Skibba, Chunlai Zeng, Xiaokun Li, Tiemin Wei, Lianpin Wu, Guang Liang

**Affiliations:** 1 Chemical Biology Research Center, School of Pharmaceutical Sciences, Wenzhou Medical University, Wenzhou, Zhejiang, China; 2 Department of Cardiology, the 5th Affiliated Hospital of Wenzhou Medical University, Lishui, Zhejiang, China; 3 Department of Cardiology, the 2nd Affiliated Hospital of Wenzhou Medical University, Wenzhou, Zhejiang, China; National Institutes of Health, UNITED STATES

## Abstract

Obesity is strongly associated with the cause of structural and functional changes of the heart in both human and animal models. Oxidative stress and inflammation play a critical role in the development of obesity-induced cardiac disorders. Curcumin is a natural product from Curcuma Longa with multiple bioactivities. In our previous study, in order to reach better anti-inflammatory and anti-oxidant dual activities, we designed a new mono-carbonyl curcumin analog, Y20, via the structural modification with both trifluoromethyl and bromine. This study was designed to investigate the protective effects of Y20 on obesity-induced cardiac injury and its underlying mechanisms. In high fat diet–fed rats, oral administration of Y20 at 20 mg/kg or curcumin at 50 mg/kg significantly decreased the cardiac inflammation and oxidative stress and eventually improved the cardiac remodeling by mitigating cardiac disorganization, hypertrophy, fibrosis and apoptosis. Y20 at 20 mg/kg showed comparable and even stronger bioactivities than curcumin at 50 mg/kg. The beneficial actions of Y20 are closely associated with its ability to increase Nrf2 expression and inhibit NF-κB activation. Taken together, these results suggest that Y20 may have a great therapeutic potential in the treatment of obesity-induced cardiac injury using Nrf2 and NF-κB as the therapeutic targets for treating obesity-related disorders.

## Introduction

An increased prevalence of obesity worldwide requires profound public health implications. Obesity is an emerging pandemic linked to type-2 diabetes mellitus, hypertension, and cardiovascular disease [1]. Evidence shows that obesity is strongly associated with structural and functional changes in the heart in both humans and animal models [2]. Presently, myocardial changes associated with the obese state is referred to as obesity cardiomyopathy which is independent of hypertension, obstructive sleep apnea and coronary artery disease [3].Mechanisms contributing to structural and functional changes in the heart due to obesity could include: altered cardiac metabolism, mitochondrial dysfunction, oxidative stress, impaired insulin signaling, inflammation, pressure/volume overload, sleep apnea, neurohumoral activation, cardiac fibrosis, and apoptosis [4]. Among these pathophysiological mechanisms, hyperlipidemia-induced inflammation and oxidative stress are the upstream indicators in the cascade and have emerged as crucial factors in obesity-induced cardiac remodeling and dysfunction [5], [6]. Therefore, antioxidant and anti-inflammatory therapies appear to be promising approaches in dealing with obesity cardiomyopathy. Several small-molecule compounds with anti-oxidant or anti-inflammatory properties have showed limited protection from obesity-induced cardiomyopathy [7,8].

**Fig 1:**
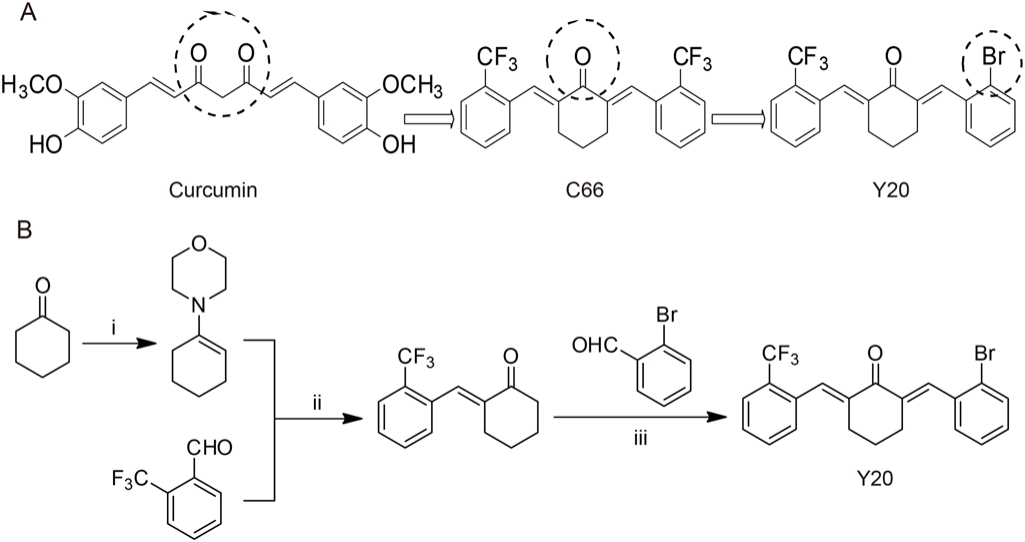
The design and synthesis of compound Y20. (A) The chemical structure of curcumin, C66 and Y20. (B) The chemical synthesis of Y20. Reagents and conditions: (i) 4-Methylbenzenesulfonic acid, toluene, 110℃ reflux, 4h; (ii) EtOH, 78℃, reflux, 5h, saturate HCl; (iii) 20% NaOH, EtOH, r.t., 10h.

Curcumin, a natural and hydrophobic polyphenol, is a constituent of the spice turmeric. It has been shown to exhibit antioxidant, anti-inflammatory, antiviral, and antibacterial activities [9]. Previous studies have demonstrated that curcumin at a dosage higher than 50 mg/kg/day can improve obesity-induced cardiac remodeling via anti-oxidative stress and anti-inflammatory mechanisms in mice [10]. Despite the favorable biological properties of curcumin, low bioavailability and instability have limited its development as a potential therapeutic drug [11]. Multiple approaches are being sought to overcome these limitations. In the past several years, our lab has focused on the chemical modification of curcumin to find novel molecules for drug development [12,13]. We have previously demonstrated that mono-carbonyl analogs of curcumin lacking the β-diketone moiety show an enhanced stability *in vitro* and an improved pharmacokinetic profile *in vivo* [14]. Of the curcumin analogs, (2E,6E)-2,6-bis(2-(trifluoromethyl) benzylidene)cyclohexanone (C66, Fig. 1A) has been shown to have the desired pharmacological effects in diabetes-related complications via its anti-inflammatory action [15,16]. However, C66 showed little anti-oxidant activity both *in vitro* and *in vivo* (data not shown), suggesting that it fails to exert the dual activities of both anti-inflammation and anti-oxidation. Thus, we desired to develop a new C66-based molecule with dual activities.

The structure of C66 has vertical symmetry and contains two trifluoromethy phenyls which may contribute to its anti-inflammatory activity. Recent studies have showed that the introduction of bromine, a radical scavenger group with anti-oxidant properties, can enhance the leading compound’s antioxidant activity [17,18]. Thus, to further modify C66 with anti-oxidant activity, we substituted one of the trifluoromethyls with a bromine, creating the new compound (2E,6E)-2-(2-bromobenzylidene)-6-(2-(trifluoromethyl)benzylidene)cyclohexanone (Y20, Fig. 1A). We hypothesized that Y20 will have anti-inflammatory properties along with anti-oxidant properties. Our previous studies demonstrated that Y20 is a safe compound without unwanted side effects when chronically administered in mice (data not shown). In the present study, we investigated whether Y20 can prevent inflammation and oxidative stress in the heart, and ultimately protect the heart from cardiac remodeling in obese rats induced by a high fat diet (HFD).

## Materials and Methods

### Chemicals and reagents

As an asymmetric mono-carbonyl curcumin analog, Y20 was prepared according to the previously reported methods [13]. The chemical synthesis was shown in Fig. 1B. Briefly, a solution of cyclohexanone (9.8 g, 0.1 mol), morpholine (10.45 g, 0.12 mol), 4-toluenesulfonic acid (0.04 g,0.23mmol) in toluene (30 ml) was under stirring for 6 h at 110°C. After completion of reaction, the solvent was removed by evaporation under reduced pressure to afford the crude product, which was used without purification. Then, 4-(cyclohex-1-en-1-yl) morpholine(4.61g, 0.027mol) and 2-(trifluoromethyl)benzaldehyde (4.0 g, 0.023 mol) in ethanol (20 ml) were added and the reaction was heated to 90°C for 6 h. After completion of reaction as indicated by TLC, the mixture was concentrated and diluted with ethyl acetate. The organic layer was separated and operated to afford the resulting crude product, which was further subjected to flash column chromatography to give (E)-2-(2-(trifluoromethyl)benzylidene)cyclohexanone. To obtain the final Y20, aqueous sodium hydroxide solution (20% w/v, 1 mL) was added to a solution of (E)-2-(2-(trifluoromethyl)benzylidene)cyclohexanone (0.40 mmol) and 2-bromobenzaldehyde (0.40 mmol) in ethanol (5 mL). The reaction mixture was stirred at room temperature for 24 h and the resulting crude product was purified by column chromatography with a 71.9% yield. The structural characterization was performed by ^1^H NMR and ESI-MS. Before Y20 was used in biological experiments, the Y20 was recrystallized from CHCl_3_/EtOH to reach HPLC purity higher than 98%. Curcumin was purchased from Sigma-Aldrich (St. Louis, MO, USA). Curcumin and Y20 were dissolved in CMC-Na (1%) for in vivo experiments. Since curcumin is of extremely high safety and the dosage of curcumin ranged from 0.5g/day to 12g/day in clinical trials [19] [20], it was usually used at a high oral dosage in animals. Here, we chose the dosage of 50mg/kg curcumin for oral administration in rats.

### Animals and treatment

The animals were obtained from the Wenzhou Medical University Animal Center. All animal procedures were performed after approval from the Ethics Committee of Wenzhou Medical University Animal Policy and Welfare Committee (Approved documents: wydw2014–0058). Twenty-four male Wistar rats (360–370 g) were randomly divided into four weight-matched groups. Six rats were fed standard animal chow and served as a normal control group (Ctrl) while the remaining 18 rats were fed with a HFD (from Medicience Diets Co. LTD, Yangzhou, China) for 12 weeks. After 8 weeks of feeding, HFD-fed rats were further divided into three groups, HFD group (n = 6), HFD treated with curcumin (n = 6), HFD treated with Y20 (n = 6). Curcumin and Y20 were given daily by gavage at a dose of 50 mg/kg or 20 mg/kg, respectively, for 4 weeks. Rats in the ND and HFD group were gavaged with a vehicle only. All the animals were provided with free access to food and water. During the experimental process, body weight and blood glucose were monitored once a week. At the end of experimental period, all the animals were euthanized by a massive pentobarbital sodium IP injection. The body weight was recorded and the blood samples were collected and centrifuged at 4°C for 10 min to collect the serum. The heart was excised aseptically, blotted dry and the weight was recorded followed by immediate freezing in liquid nitrogen and stored at -80°C for further analysis.

### Histological analyses

Excised heart tissue specimens were fixed in 4% formalin, processed in graded alcohol, xylene, and then embedded in paraffin. Paraffin blocks were sliced into 5 μm sections. After rehydration, the sections were stained with Hematoxylin and Eosin (H&E), sirius red, and masson’s trichrome. To evaluate the histopathological damage, each image of the sections was captured using a light microscope (400×amplification, Nikon, Japan). To detect apoptosis, tissue sections were used for the terminal deoxynucleotidyl transferase-mediated dUTP nick end labeling (TUNEL) apoptosis detection kit (R&D Systems, Minneapolis, MN) according to the manufacturer’s instruction. TUNEL positive cells were imaged under a fluorescence microscope (400×amplification, Nikon, Japan).

### Immunohistochemical analysis

The paraffin samples (5 μm) were removed from the sections with xylene, rehydrated in graded alcohol series, subjected to antigen retrieval in 0.01 mol/L citrate buffer (pH 6.0) by microwaving, and then placed in 3% hydrogen peroxide in methanol for 30 min at room temperature. After blocking with 5% BSA, the sections were incubated with anti-3-NT antibody (1:500, Abcam Inc, MA), anti-TNF-α antibody (1:500, Abcam Inc, MA) or anti-CD68 (1:200, Santa Cruz, CA, USA), respectively, overnight at 4°C, followed by the respective secondary HRP-conjugated antibody (Santa Cruz, CA, USA). After counterstaining with hematoxylin, the sections were dehydrated and viewed under the Nikon microscope (400×amplification, Nikon, Japan).

### Immunofluorescence staining of tissues

The paraffin samples (5 μm) were removed from the sections using xylene, rehydrated in graded alcohol series, subjected to antigen retrieval in 0.01 mol/L citrate buffer (pH 6.0) by microwaving, and then placed in 3% hydrogen peroxide in methanol for 30 min at room temperature. After blocking with 5% BSA, the sections were incubated with the antibody for nuclear factor erythroid 2-related factor 2 (Nrf2) (Santa Cruz, CA, USA), or antibody for tumor necrosis factor (TNF)-α (Abcam Inc, MA) overnight at 4°C, followed by FITC-conjugated secondary antibody (1:200, Santa Cruz, CA, USA). The nucleus was stained with DAPI and sections were then viewed under a Nikon fluorescence microscope (400×amplification, Nikon, Japan).

### Measurements of the level of serum lipid

The components of serum lipid including the total triglyceride (TG), total cholesterol (TCH), low density lipoprotein (LDL), and high density lipoprotein (HDL) were detected using commercial kits (Nanjing Jiancheng Bioengineering Institute, Jiangsu, China).

### Real-time quantitative PCR

Total RNA was isolated from the tissues (50–100 mg) using TRIZOL (Invitrogen, Carlsbad, CA) according to the manufacturer’s instructions. Reverse transcription and quantitative PCR were performed using M-MLV Platinum RT-qPCR Kit (Invitrogen, Carlsbad, CA). Real-time qPCR was carried out using the Eppendorf Real plex 4 instrument (Eppendorf, Hamburg, Germany). Primers for genes including TNF-α, interleukin (IL)-6, IL-1β, cyclooxygenase 2 (COX-2), Nrf2, Heme oxygenase 1 (HO-1), NAD(P)H quinone oxidoreductase 1 (NQO-1), Transforming growth factor (TGF)-β, collagen 1, matrix metalloproteinase (MMP)-2, MMP-9, atrial natriuretic peptide (ANP), brain natriuretic peptide (BNP), Intercellular Adhesion Molecule 1 (ICAM-1), vascular cell adhesion molecule 1 (VCAM-1), and β-actin were synthesized by Invitrogen (Invitrogen, Shanghai, China). The primer sequences used were shown in Table 1. The relative amount of each gene was normalized to the amount of β-actin.

**Table 1:** Primer sequences for real-time quantitative PCR.

Gene	Species	Forward primer	Reverse primer
ICAM-1	Rat	AGATCATACGGGTTTGGGCTTC	TATGACTCGTGAAAGAAATCAGCTC
VCAM-1	Rat	TTTGCAAGAAAAGCCAACATGAAAG	TCTCCAACAGTTCAGACGTTAGC
MMP9	Rat	CCCCACTTACTTTGGAAACGC	ACCCACGACGATACAGATGCTG
MMP2	Rat	GACCTTGACCAGAACACCATCG	GCTGTATTCCCGACCGTTGAAC
TGF-β	Rat	GGACTACTA CGCCAA AGA AG	TCA AAAGACAGCCACTCAGG
Collagen1	Rat	GAGCGGAGAG TACTGGATCGA	CTGACCTGTCTCCAT- GTTGCA
ANP	Rat	CTGCTAGACCACCTGGAGGA	AAGCTGTTGCAGCCTAGTCC
BNP	Rat	GATCCAGGAGAGACTTCGAAA	CGGTCTATCTTCTGCCCAA
IL-1β	Rat	CACCTCTCAAGCAGAGCACAG	GGGTTCCATGGTGAAGTCAAC
TNF-α	Rat	TACTCCCAGGTTCTCTTCAAGG	GGAGGCTGACTTTCTCCTGGTA
IL-6	Rat	GAGTTGTGCAATGGCAATTC	ACTCCAGAAGACCAGAGCAG
Cox2	Rat	CGGAGGAGAAGTGGGGTTTAGGAT	TGGGAGGCACTTGCGTTGATGG
Nrf2	Rat	TTCCTCTGCTGCCATTAGTCAGTC	GCTCTTCCATTTCCGAGTCACTG
HO-1	Rat	TCTATCGTGCTCGCATGAAC	CAGCTCCTCAAACAGCTCAA
NQO-1	Rat	ACTACGATCCGCCCCCAACTTCTG	CTTCGGCTCCCCTGTGATGTCGT
β-actin	Rat	ATCGTGGGCCGCCCTAGGCACC	CTCTTTAATGTCACGCACGATTTC

### Western blot assay

Primary antibodies for B-cell lymphoma 2 (Bcl-2), Bcl-2-associated X protein (Bax), ANP, IκBα, VCAM-1, TGF-β, cleavaged PARP, GAPDH and secondary antibodies were purchased from Santa Cruz Biotechnology (Santa Cruz, CA). Tissue lysate homogenates were prepared in our lab. Protein samples (30–80 μg) were subjected to a 10% sodium dodecyl sulfate-polyacrylamide gel electrophoresis and transferred onto polyvinyldene fluoride membrane (Bio-Rad Laboratory, Hercules, CA). After blocked in blocking buffer (5% milk in tris-buffered saline containing 0.05% Tween 20, TBS-T) for 1.5 h at room temperature, the membranes were incubated with different primary antibodies overnight at 4°C. The membranes were then washed in TBS-T and reacted with secondary horseradish peroxidase-conjugated antibody for 1–2 h at room temperature. Antigen-antibody complexes were then visualized using enhanced chemiluminescence reagents.

### Statistical analysis

Data was presented as means ± SDs. The statistical significance of differences between groups was obtained using the student’s t test or ANOVA multiple comparisons in GraphPad Pro 5.0 (GraphPad, San Diego, CA). Differences were considered to be significant at P < 0.05.

## Results

### The effects of Y20 on body weight, blood glucose, and the serum lipid profile

**Fig 2:**
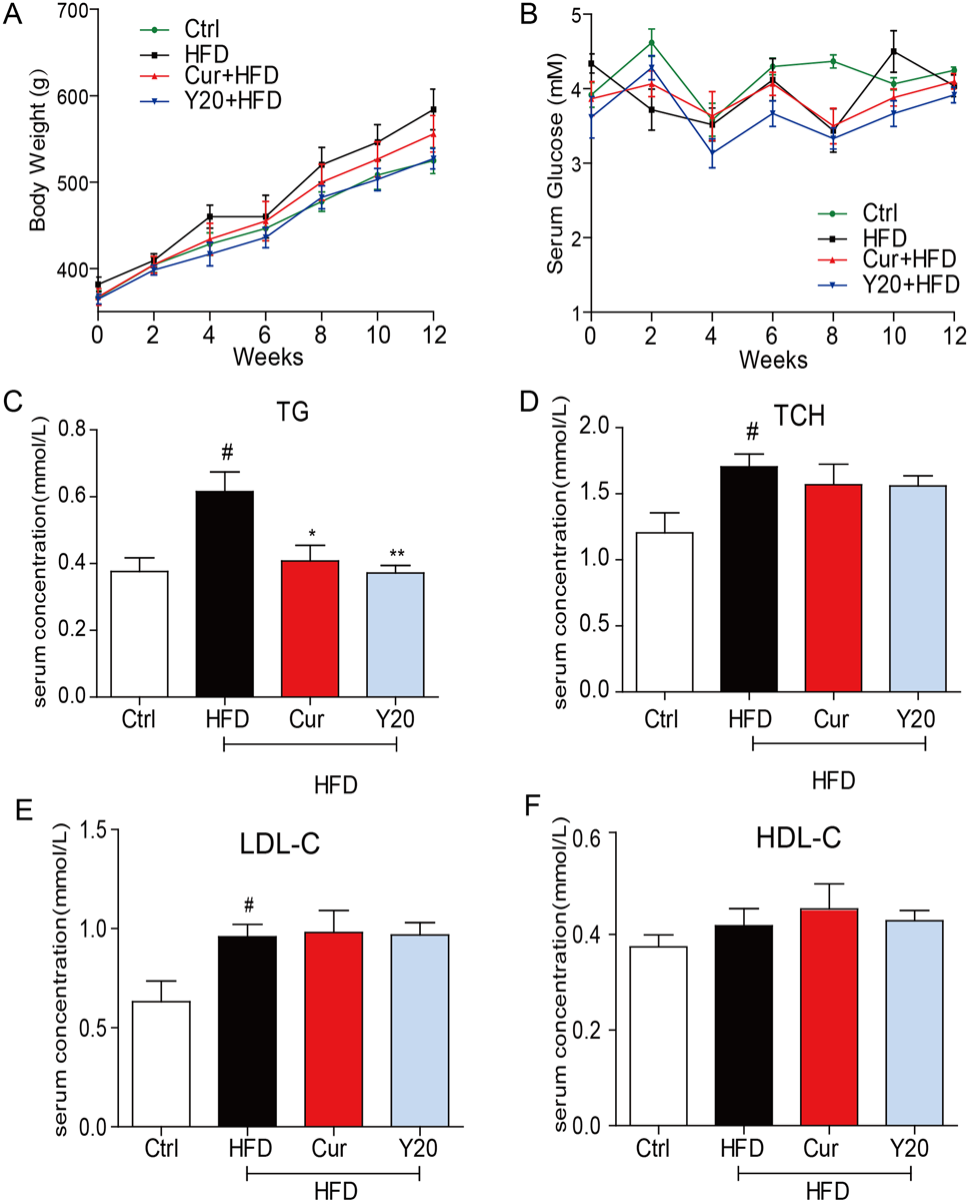
The effects of Y20 and curcumin treatment on the profiles of body weight, blood glucose and serum lipids in HFD-fed rats. Male Rats weighted at 360 g-370 g were firstly fed either a normal diet (Ctrl) or high fat diet (HFD) for 8 weeks and then the HFD-fed rats were orally treated with curcumin (Cur, 50 mg/kg), Y20 (20 mg/kg) or vehicle (1% CMC-Na) every day for 4 weeks (n = 6 in each group). In this process, the body weight and blood glucose were monitored once every week. At the end of experiment, the rats were sacrificed and the blood samples were collected and centrifuged for collecting serum for serum lipids analysis. (A) The body weight; (B) The blood glucose; (C) The level of serum triglycerides (TC); (D) The level of serum total cholesterol (TCH); (E) The serum level of low density lipoprotein cholesterol (LDL-C); (F) The level of serum high density lipoprotein cholesterol (HDL-C); data are presented as mean±SDs, n = 6; # *P*<0.05 v.s. vehicle control (Ctrl); *P<0.05; **P<0.01 v.s. HFD group.

HFD-fed rats became markedly obese with a body weight above 550 g on average at the 12-week time point, treatment with curcumin (50 mg/kg) mildly induced weight loss in HFD-fed rats at the 12-week timet point (*P*> 0.05 v.s. HFD group), while treatment with Y20 (20 mg/kg) markedly reduced the body weight gain in HFD-fed rats at the 12-week point (*P*< 0.05 v.s. HFD group, Fig. 2A). HFD-fed rats did not exhibit significant changes in the level of blood glucose. Also, treatment with curcumin or Y20 had no effect on the blood glucose in HFD-fed rats (Fig. 2B). In the basal fasting state, HFD-fed rats exhibited a significant increase in the levels of serum lipid including triglycerides (TG) (Fig. 2C), total cholesterol (TCH) (Fig. 2D) and low density lipoprotein cholesterin (LDL-C) (Fig. 2E), but no significant change in high density lipoprotein cholesterin (HDL-C) (Fig. 2F). Treatment with curcumin (50 mg/kg) or Y20 (20 mg/kg) significantly decreased the increase in TG level (*P*<0.05 v.s. HFD group, Fig. 2C), while neither curcumin nor Y20 had an effect on the TCH, LDL-C, and HDL-C levels in HFD rats (Fig. 2D-2F).

### Y20 attenuates HFD-induced myocardial inflammation

**Fig 3:**
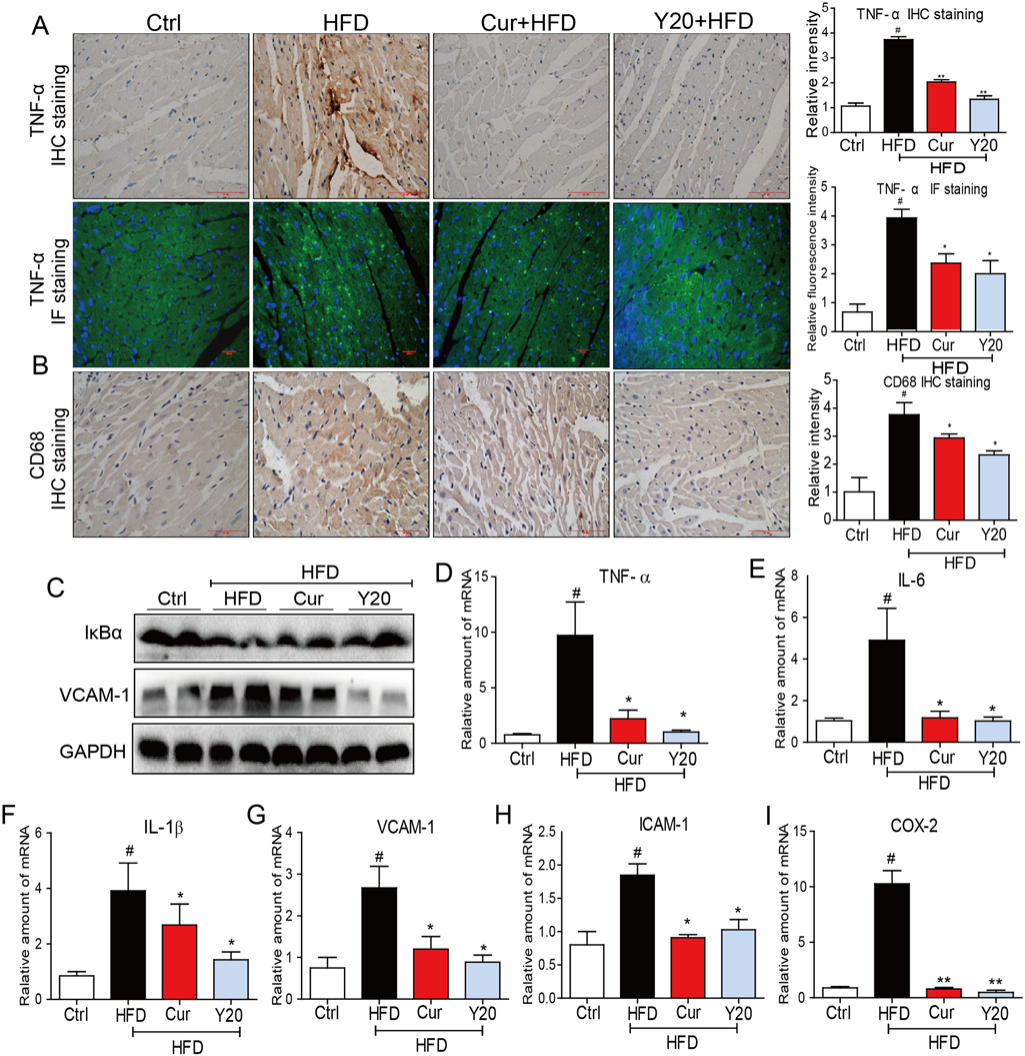
Y20 attenuates cardiac inflammation in the hearts of HFD-fed rats. (A) Representative images for immunohistochemical staining and immunofluorescent staining of TNF-α accumulation using the formalin-fixed myocardial tissues as described in Methods (400×magnification). The statistic data of the relative intensity was determined by imageJ software (*NIH*, *Bethesda*, *MD*), and data are presented as mean±SDs, n = 4; (B) Representative images for immunohistochemical staining of CD68 expression using the formalin-fixed myocardial tissues as described in Methods (400×magnification). The statistic data of the relative intensity was determined by imageJ software (*NIH*, *Bethesda*, *MD*), and data are presented as mean±SDs, n = 4; (C) Western blot analysis for the protein expression of IκB-α and VCAM-1 in the myocardial tissues was performed. (D-I) The mRNA expression of TNF-α, IL-6, IL-1β, VCAM-1, ICAM-1 and COX-2 in myocardial tissues was detected by real-time qPCR. Four rats in each group were used for above analysis. * *P*<0.05, ** *P*<0.01 v.s. HFD group; # *P*<0.05 v.s. vehicle control (Ctrl).

We then detected the inflammatory markers in cardiac tissues. TNF-α immunohistochemistry (IHC) staining and immunofluorescent (IF) staining showed that there was a significant increase in TNF-α protein accumulation (TNF-α were stained in brown for IHC, and in green for IF) in the hearts of HFD-fed rats (Fig. 3A). There was also an increased expression of CD68, a specific marker for macrophages, in the hearts of the HFD group, suggesting inflammatory cell infiltration in the obese hearts (CD68 positive cells were stained in brown, Fig. 3B). VCAM-1, a critical cell adhesion molecule for inflammatory cell infiltration, was then found to be increased in the hearts of the HFD group as evidenced by western blot analysis (Fig. 3C). In addition, IκB degradation was observed in the hearts of the HFD-fed rats, indicating nuclear factor κB (NF-κB), a transcript factor controlling the expression of a variety of pro-inflammatory cytokines, was activated via hyperlipidemia (Fig. 3C). We then determined the mRNA expression of several inflammatory genes by real-time qPCR. The results in Fig. 3D-3I showed that the expression of pro-inflammatory cytokines such as TNF-α, IL-6, IL-1β and COX-2 and cell adhesion molecules such as VCAM-1 and ICAM-1 were all up-regulated in myocardial tissues of obese rats. However, all of the above increases in cardiac inflammation were significantly attenuated by 4-week treatment with either curcumin or Y20; and generally, Y20 at 20 mg/kg exhibited a slightly stronger anti-inflammatory activity than curcumin at 50 mg/kg (Fig. 3A-3I).

### Y20 attenuates HFD-induced myocardial oxidative stress

**Fig 4:**
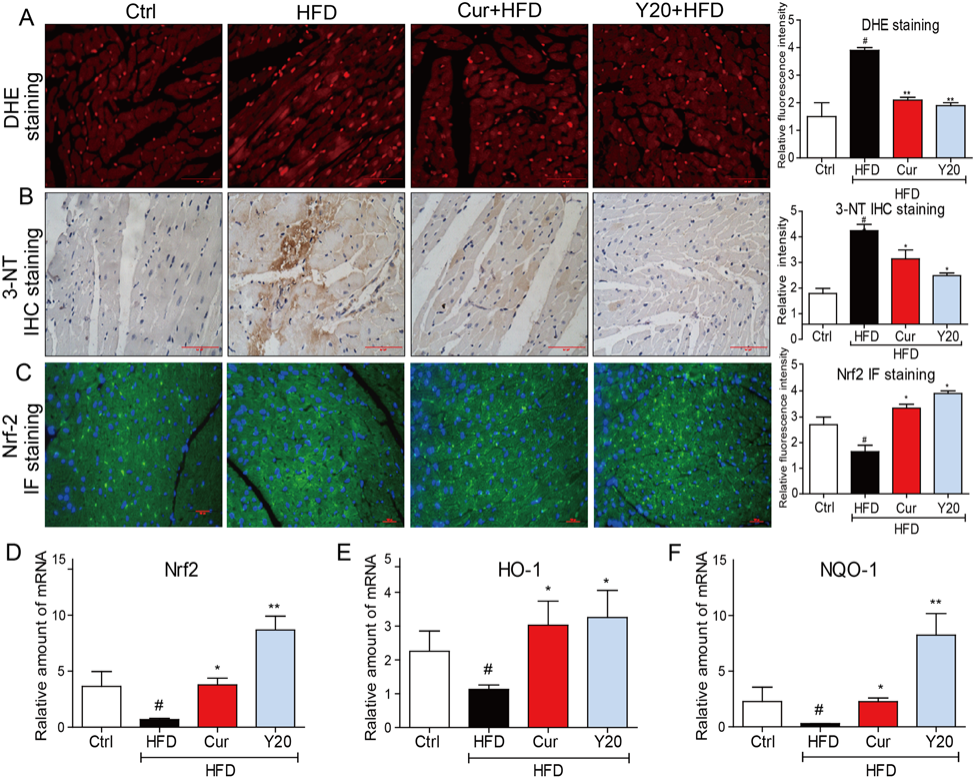
Y20 attenuates HFD-induced myocardial oxidative stress. (A) Representative images for DHE staining using the frozen section of heart tissues as described in Methods (400×magnification).The statistic data of the relative intensity was determined by imageJ software (NIH, Bethesda, MD), and data are presented as mean±SDs, n = 4; (B) Representative images for immunohistochemical staining of 3-NT accumulation using the formalin-fixed myocardial tissues as described in Methods (400×magnification). The statistic data of the relative intensity was determined by imageJ software (NIH, Bethesda, MD), and the date are presented as mean±SDs, n = 4; (C) Representative images for immunofluorescent staining of Nrf2 using the formalin-fixed myocardial tissues as described in Methods (400×magnification). The statistic data of the relative intensity was determined by imageJ software (NIH, Bethesda, MD), and the date are presented as mean±SDs, n = 4; (D-F) The mRNA expression of Nrf2, HO-1 and NQO-1 in myocardial tissues was detected by real-time qPCR. Data are presented as mean±SDs, n = 4. * P<0.05, ** P<0.01 v.s. HFD group; # P<0.05 v.s. vehicle control (Ctrl).

We investigated whether Y20 administration can prevent oxidative stress induced by HFD feeding in rat hearts. Immunohistochemistry and immunofluorescent staining analysis showed that the superoxide anion production (stained in red, Fig. 4A) and 3-NT (stained in brown, Fig. 4B) in the heart tissues of HFD group were significantly enhanced. However, either curcumin (50 mg/kg) or Y20 (20 mg/kg) significantly prevented HFD-induced cardiac accumulation of these two oxidative stress markers. Evidence has demonstrated that curcumin could up-regulate and activate Nrf2, a key transcription factor in the regulation of multiple antioxidants. [21] Thus, we investigated whether Y20 had the same effects on Nrf2 expression in myocardial tissues. Fig. 4C and 4D showed that HFD feeding down-regulated the expression of Nrf2 at both the protein and mRNA levels, while Nrf2 expression was significantly increased with treatment of either curcumin or Y20. Similar results were observed in the profile of Nrf2 downstream genes such as HO-1 (Fig. 4E) and NQO-1 (Fig. 4F). Interestingly, Y20 at 20 mg/kg was more efficient than curcumin at 50 mg/kg in both reducing reactive oxygen species (ROS) and up-regulating Nrf2 expression.

### Y20 treatment attenuates HFD-induced cardiac histopathology and hypertrophy

**Fig 5:**
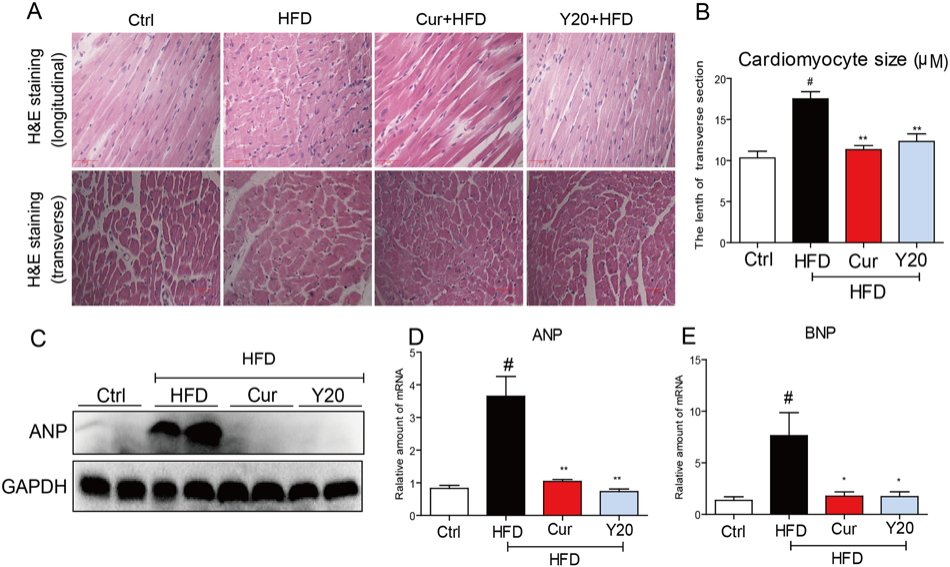
Y20 attenuates cardiac histological abnormalities and hypertrophy in the hearts of HFD-fed rats. (A) Representative images for the Hematoxylin-Eosin (H&E) staining in the formalin-fixed myocardial tissues (400× magnification). (B) Qantitative data of myocyte cross-section lenth of 100 cells chosen from different visual scopes of 4 samples per group in myocardial transverse H&E staining were shown. (C) Western blot analysis for the protein expression of ANP in the myocardial tissues was performed. (D & E) The mRNA expression of the hypertrophic markers ANP and BNP in the myocardial tissues was detected by real-time qPCR. Four rats in each group were used for above analysis. *, *P*<0.05, **, *P*<0.01 v.s. HFD; # *P*<0.05 v.s. vehicle control (Ctrl).

The myocardial structural was examined by H&E staining. As shown in Fig. 5A, the cardiac longitudinal section in the H&E staining exhibited well organized myofibrils and disorganized fibres in the control rats and in HFD-fed hearts, respectively. Curcumin or Y20 treatment reduced the histopathological alterations in the myofibril organization of the HFD hearts (Fig. 5A). Cardiac transverse section H&E staining showed that the cardiomyocyte diameter was significantly increased in the HFD group, while curcumin or Y20 administration significantly attenuated the HFD-induced cardiomyocyte hypertrophy (Fig. 5A). Cell size measurements from the transverse section confirmed a significant reduction in the cardiaomyocyte hypertrophy by curcumin and Y20 (Fig. 5B). Consistent with the morphologic observations, the protein expression of the cardiac hypertrophic marker ANP was significantly increased in HFD-fed rats, which were reversed by cucumin or Y20 treatment (Fig. 5C). Real-time PCR analysis further confirmed similar results in the level of mRNA transcription of ANP and BNP (Fig. 5D & 5E).

### Y20 attenuates HFD-induced cardiac fibrosis

**Fig 6:**
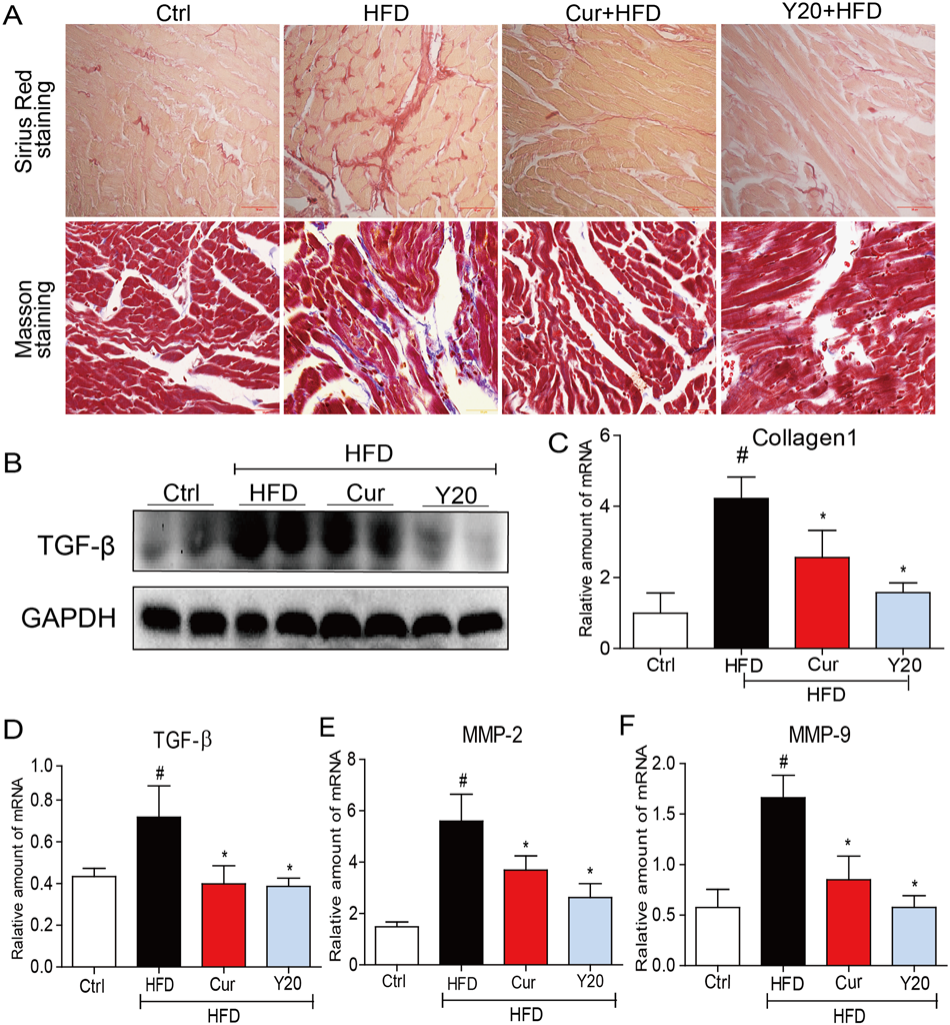
Y20 attenuates cardiac fibrosis in the hearts of HFD-fed rats. (A) Representative images for Sirius Red staining and masson staining in the formalin-fixed myocardial tissues indicating collagen deposition and implying the extent of cardiac fibrosis (400×magnification). (B) Western blot analysis for the protein expression of TGF-β in the myocardial tissues was performed. (C-F) The mRNA expression of fibrotic markers such as collagen 1, TGF-β, MMP-2 and MMP-9 in the myocardial tissues was detected by real-time qPCR. Four rats in each group were used for above analysis. * *P*<0.05, ** *P*<0.01 v.s. HFD group; # *P*<0.05 v.s. vehicle control (Ctrl).

Myocardial fibrosis was examined by Sirius Red staining and Masson’s trichrome staining, respectively. Increased collagen type I synthesis and deposition contribute to enhancement of myocardial fibrosis [22]. As shown in Fig. 6A, increased collagen 1 and fibrosis were observed in the HFD-induced hearts. However, these fibrotic changes were significantly attenuated in the curcumin and Y20 treated groups. Western blot analysis revealed a significant increase in the pro-fibrotic gene, TGF-β expression in the hearts of HFD-fed rats (Fig. 6B); real-time qPCR assay showed marked increases in the expression of pro-fibrotic genes including collagen 1, TGF-β, MMP-2, and MMP-9 in the hearts of HFD-fed rats (Fig. 6C-6F). Either curcumin (50 mg/kg) or Y20 (20 mg/kg) treatment for 4 weeks significantly attenuated HFD-induced expression of these genes (Fig. 6C-6F). In general, Y20 displays a stronger anti-fibrosis activity than curcumin.

### Y20 attenuates HFD-induce myocardial apoptosis

**Fig 7:**
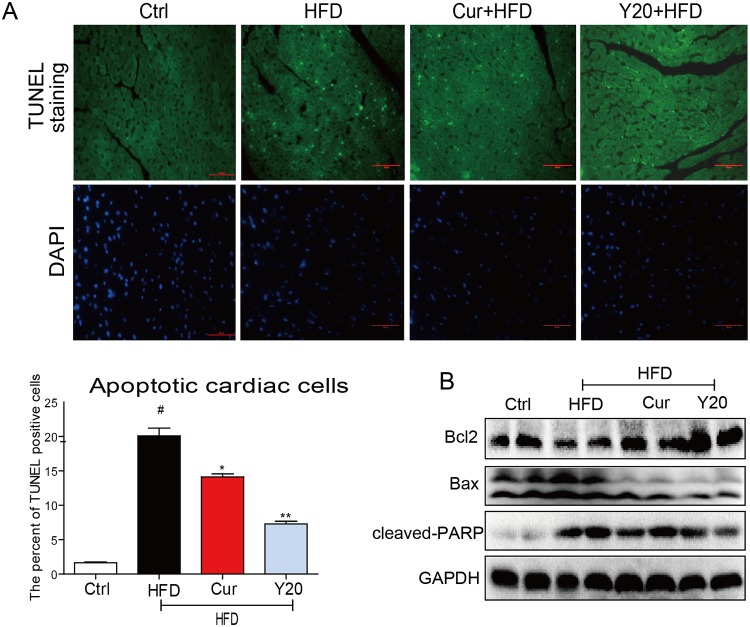
Y20 mitigates HFD-induced cardiac apoptosis in HFD-fed rats. (A) Representative images and statistic figure for TUNEL staining in heart tissues sections. (B) Western Blot analysis for the protein expression of apoptosis-related proteins in myocardial tissues was performed. Four to six rats in each group were used for above analysis. * *P*<0.05, ** *P*<0.01 v.s. HFD group; # *P*<0.05 v.s. vehicle control (Ctrl).

Cardiomyocyte apoptosis is a comprehensive consequence of myocardial abnormalities.[23] As shown in Fig. 7A, apoptotic cells stained in bright green by TUNEL staining, were remarkably increased in HFD-induced rat hearts, while either curcumin or Y20 treatment significantly reversed HFD-induced myocardial cell apoptosis. Y20 at 20 mg/kg also showed stronger anti-apoptotic ability than curcumin at 50 mg/kg. The expression of anti-apoptotic protein Bcl-2 and pro-apoptotic protein Bax were assessed by western blot analysis. Enhanced expression of Bax and reduced expression of Bcl-2 was observed in HFD group, both of which were significantly reversed by curcumin or Y20 administration (Fig. 7B). In addition, similar results were observed when examining the level of cleaved PARP, a marker of cell apoptosis (Fig. 7B).

## Discussion

The incidence of obesity is increasing worldwide and considered a major public health concern. Researches on ways to slow the development of obesity have traditionally focused on dietary and lifestyle modifications such as restricting caloric intake and increasing physical activity [24]. Obesity is strongly associated with structural and functional changes in the heart in both humans and animal models [2]. Cardiac consequences of obesity include cardiac remodeling such as cardiac hypertrophy, cardiac fibrosis, cardiac apoptosis and subclinical impairment of LV systolic and diastolic function [4,25]. As observed in our study, HFD feeding for 12 weeks significantly increased the rat body weight and hyperlipidemia (Fig. 2). Under a HFD regimen, cardiac remodeling including cardiomyocyte disorganization, hypertrophy, fibrosis and apoptosis were evident in the hearts of obese rats (Figs. 5–7), accompanied by significantly increased cardiac inflammation and oxidative stress (Figs. 3–4). These results are consistent with previous reports that the mechanisms underlying the obesity-induced cardiac remodeling is multifactorial using several mechanisms such as inflammation and oxidative stress [4].

Studies to prevent or reverse HFD-induced cardiac pathophysiology are of high interest due to their translation to the clinical setting for the prevention of cardiac disorders. Our data collectively favor a unique role of a newly designed curcumin analog, Y20, against HFD-induced heart injury, possibly through the mechanisms associated with reduced inflammation and oxidative stress.

Curcumin has been used in a variety of chronic diseases. Growing evidence has suggested that curcumin can be used to treat obesity and obesity-related metabolic diseases because it can reverse insulin resistance, hyperglycemia, hyperlipidemia, and inflammatory symptoms associated with obesity and metabolic diseases [26]. It is also reported that curcuminoid can protect from obesity-induced cardiac injury [27]. However, due to the chemical instability and poor bioavailability, the clinical application of curcumin has been significantly limited [11] Our lab previously designed a series of mono-carbonyl analogs of curcumin (MACs), which showed enhanced stability in vitro and improved pharmacokinetic profile in vivo [14]. Among these MACs, an analog, C66, has been found to be able to attenuate diabetic nephropathy and cardiomyopathy via an anti-inflammatory mechanism [15,16]. In order to further confer C66 with anti-oxidant activity, an antioxidant modification of C66 has been performed via the introduction of a bromine atom, which has been applied in the structures of a number of anti-oxidant agents [17,18]. Thus, a new derivative, Y20 was developed with the hopes of having both anti-inflammatory and anti-oxidant activities. Y20 showed both anti-inflammatory and anti-oxidant effects when applied in vivo, even slightly stronger than curcumin (Figs. 3 and 4). More importantly, Y20 was able to have the desired anti-inflammatory and anti-oxidative effects when the dosage was 2.5-fold lower than that of curcumin, which may attribute to the improvement of the pharmaceutical profile of Y20 as one of the MACs. Although Y20 is not significantly different from curcumin in vivo when examining the present figures and data, the dosage of Y20 used here is 2.5-fold lower than that of curcumin. Thus, Y20 may be a more promising candidate than curcumin in cardioprotection both pharmacokinetically and pharmacologically. Further studies should be performed to test the in vitro anti-inflammatory and anti-oxidative effects and in vivo pharmacokinetic profile of Y20.

A large body of evidence suggests that obesity is associated with a low-grade chronic inflammation characterized by overproduction of pro-inflammatory cytokines and infiltration of monocyte/macrophages in the kidney, heart, liver, and adipose tissues [28,29]. Here, we also found that there was increased accumulation of cytokines and macrophages in the obese hearts, which matches with the previous studies [30]. These results suggested that the obesity causes inflammation in cardiomyopathy. Studies have demonstrated a pivotal role of various cytokines in the myocardial injury by directly inducing fibrosis, hypertrophy, apoptosis, and ultimately contractility [31,32]. Interestingly, all these abnormalities were reversed by treatment with curcumin or Y20. The anti-inflammatory activity of Y20 was closely associated with its cardiac protective effects.

Degradation of IκBα mediates the transcription factor NF-κB activation. NF-κB has a central role in the regulation of inflammation and has been demonstrated to be involved in the pathogenesis of obesity-related cardiovascular diseases [33,34]. Thus, NF-κB may be considered a therapeutic target in obesity-induced cardiac inflammation or cardiac injury. Curcumin has been reported to inhibit NF-κB activation in a variety of cell lines [35,36]. Interestingly, our study showed that the analog Y20 also efficiently reversed IκB degradation. Therefore anti-inflammatory effect of Y20 may be due to NF-κB inhibition.

Evidence has suggested that obesity could result in cardiac oxidative stress [37]. Furthermore, increased oxidative stress has been linked with cardiac hypertrophy, fibrosis, contractile dysfunction, and heart failure [38]. Meanwhile, pharmacological antioxidants have been shown to improve obesity-induced cardiomyopathy [39], indicating the independent involvement of oxidative stress in the pathogenesis of cardiomyopathy in obesity. Superoxide anion (O_2_^-^) production and 3-NT accumulation were increased in the hearts of HFD-fed rats, indicating an obesity-induced oxidative stress and high ROS levels in the obese hearts (Fig. 4). Y20 treatment in obese rats significantly reversed HFD-induced cardiac oxidative stress, which contributes to its cardioprotective effects. Our studies also revealed Y20 increases the expression of Nrf2 and the downstream antioxidant genes such as HO-1 and NQO-1. Nrf2, as a master regulator of cellular defense against oxidative stress, has been reported to be a promising target to treat obesity [40]. Previous studies already suggested there was a reduced expression of Nrf2 in the heart, skeletal muscle, and liver in HFD-fed animals [41,42]. Curcumin has also been found to up-regulate Nrf2 expression and activity in cellular and animal models [21]. Y20 treatment significantly enhanced Nrf2 expression, as well as Nrf2 activity as revealed by increased levels of HO-1 and NQO-1, in the hearts of HFD-fed rats. It is speculated that the anti-oxidative properties of Y20 may result from the Nrf2 activation. This study also suggests that attenuating oxidative stress may be a therapeutic strategy in treating obesity-induced heart injury.

In conclusion, the findings of the present study demonstrated the defensive role of a newly designed curcumin analog, Y20, against oxidative stress, inflammation, apoptosis, hypertrophy and fibrosis in obesity-induced heart injury. Although the reduced body weight gain and serum triglyceride increase may partially contribute to the beneficial effects of Y20 in obesity cardiomyopathy, the beneficial actions of Y20 are closely associated with its ability to increase Nrf2 and inhibit NF-κB. This clearly suggests that Y20 could be used for therapeutic application in the treatment of obesity-related cardiac disorders. In addition, Nrf2 and NF-κB, for the regulation of oxidative stress and inflammation, respectively mediate the hypertrophic, apoptotic, and fibrotic effects in HFD-induced hearts. These results may provide a deeper understanding of the mechanism and treatment of hyperlipidemia-induced cardiac injury and obesity-related disorders.
